# Analysis of cortical dysplasias using b-tensor encoding diffusion MRI in an animal model

**DOI:** 10.1371/journal.pone.0346132

**Published:** 2026-04-03

**Authors:** Olimpia Ortega-Fimbres, Ricardo Ríos-Carrillo, Edith Gaspar-Martínez, Priscila Ruiz-Acosta, Mirelta Regalado, Hiram Luna-Munguía, Alonso Ramírez-Manzanares, Luis Concha

**Affiliations:** 1 Instituto de Neurobiología, Universidad Nacional Autónoma de México, Querétaro, México; 2 Centre for Functional and Metabolic Mapping, Robers Research Institute, Western University, London, Ontario, Canada; 3 Departamento de Ciencias de Computación, Centro de Investigación en Matemáticas, A.C., Guanajuato, México; Medical Center - University of Freiburg, GERMANY

## Abstract

Cortical dysplasias are malformations of cortical development characterized by disorganization of the cyto- and myeloarchitecture of the neocortex. They are a common cause of epilepsy and their diagnosis through conventional imaging can often be challenging, hindering surgical treatments. Diffusion-weighted magnetic resonance imaging (dMRI) has the ability to infer tissue properties at the microscopic scale, making it a promising technique for detection of cortical dysplasias. This study aims to assess the microarchitecture of the cerebral cortex in a murine model of cortical dysplasia using dMRI acquired with b-tensor encoding. Pregnant Sprague-Dawley rats were administered either carmustine (BCNU) or saline solution on day 15 of gestation. Their offspring were imaged at 120 days of age using a 7 tesla scanner, acquiring diffusion-sensitive images with b-tensor encoding. Images were processed with Q-space trajectory imaging with positivity constraints (QTI+) to derive various metrics along a curvilinear coordinate system across the neocortex. After scanning, the brains were processed for immunofluorescence and histological examinations. Experimental animals exhibited a significant reduction of microscopic fractional anisotropy (µFA) and anisotropic kurtosis (K_shear_) in the middle and lateral cortical layers compared to the control animals. Immunofluorescence and histological analysis showed decreased and dysorganized myelinated fibers, and an increase of glial processes in BCNU-treated animals. Given the applicability of b-tensor encoding in clinical scanners, this approach holds promise for improving detection of focal cortical dysplasias in patients with epilepsy.

## 1 Introduction

The cerebral cortex exhibits a highly organized architecture. Alterations during prenatal neurodevelopment can lead to diverse anatomical abnormalities that vary in extent and severity as a consequence of the precipitating insult. Focal cortical dysplasias (FCDs) are a specific type of malformation of cortical development, representing the first and second cause of pharmacoresistant focal epilepsy in children and adults, respectively [1]. These malformations are characterized by disrupted cortical layers, neuronal heterotopia, and presence of dysmorphic neurons [2]. Magnetic resonance imaging (MRI) is the primary diagnostic tool used for detection of FCDs and to guide surgical interventions, when appropriate [3]. Diagnosis of FCDs can be challenging, as their detection relies on subtle visual cues such as focal cortical thickening, slight hyperintensity, and blurring of the gray/white matter boundary on conventional T1- or T2-weighted MRI [4]. Moreover, there can be ample variation in their size and anatomical location [5–7]. This results in frequent underdiagnosis and inadequate treatment, highlighting the need for more sensitive and specific imaging methods [4]. Detection of FCDs can be improved substantially through quantitative methods that augment the diagnostic yield of conventional MRI, such as texture analysis [8], voxel-based morphometry [9], and artificial intelligence [10,11], and more recently through acquisition and analysis of MRI fingerprinting [12,13].

Given that anatomical abnormalities that usually accompany FCDs can be subtle, there is a need for imaging methods that are able to capture the histopathological features that characterize these lesions. Diffusion-weighted MRI (dMRI) offers an alternative, non-invasive approach for studying tissue microarchitecture by measuring the diffusion of water molecules in different tissues [14–16]. This technique can capture structural details that are not visible with conventional MRI, making it potentially more effective for diagnosing FCD [17]. Furthermore, dMRI can be applied to both human and animal models, enabling the translation of experimental findings to clinical practice [18,19]. Several methods to analyze the diffusion signal have been introduced, the majority of which are implemented on data acquired using single diffusion encoding [20]. While these methods have been mainly used to characterize white matter, they can also provide relevant information regarding the laminar and columnar structure of the human, non-human primate, and rodent neocortex [21–27]. Recent advances in dMRI acquisition and analysis methods have further improved the diagnostic capacity of this technique by providing more information on tissue characteristics [28,29]. Multidimensional encoding techniques have been developed to allow a more thorough analysis of brain microstructure by encoding diffusion through complex, time-varying gradient waveforms [30,31]. In conventional single diffusion encoding, diffusion weighting is applied using gradients of various orientations and magnitudes (b-values), and the encoding can be fully described by a single vector. In multidimensional encoding, this concept is generalized: the diffusion-encoding vector is replaced by a second-order tensor (the b-tensor), allowing control not only over orientation and magnitude, but also over the shape of the diffusion encoding. Different b-tensor shapes (e.g., linear, planar, or spherical) query the tissue in complementary ways and yield information not reachable by single diffusion encoding. Indeed, while diffusion tensor imaging (DTI) provides characteristics of a single diffusion tensor per voxel, b-tensor encoding adds a metric of the covariance of domain D-tensors, which allows sampling properties of intra-voxel diffusion tensor distribution and provides rich information that better accounts for the heterogeneity of nervous tissue components [32]. These innovations make b-tensor encoded dMRI a promising tool for characterizing the microarchitecture of the cortex and alterations present in FCDs and other cortical malformations [33].

In this study, we used advanced dMRI techniques to characterize tissue properties of the cortex in an animal model of FCD. Histological analyses were performed to bridge tissue properties with water diffusion patterns in the regions of interest. The aim was to assess whether b-tensor encoded dMRI methods are sensitive to the histopathological features of FCD as an initial exploration of their clinical applicability.

## 2 Methods

All experimental procedures were approved by the Ethics Committee of the Institute of Neurobiology (Universidad Nacional Autónoma de México; protocol 111-A).

### 2.1 Murine model of cortical dysplasia

To induce the histopathological features of FCD Type IIa in rodents, we used a known animal model that disrupts corticogenesis *in utero*. Sprague-Dawley rats were injected with a single dose of either the alkylating agent BCNU (bis-chloroethylnitrosourea, also known as carmustine; 20 mg/kg in saline solution, i.p., 5; 5 rats), or saline solution (for control; 3 rats) on embryonic day 15; a time point that corresponds to the peak of cortical neurogenesis [34]. Based on previous reports [35,36] and our observations [27,37], this procedure is known to induce cortical alterations in the pups similar to those found clinically [1,38]. The pups were kept with their mothers until weaning. All animals had access to food and water *ad libitum* and were always kept at the animal facility under controlled environmental conditions. Experiments were only conducted with the offspring. A total of 18 control (6 female) and 20 BCNU-treated (8 female) rats were included for further analysis. None of the animals died, nor displayed any signs of poor quality of life, suffering, or distress throughout the study.

### 2.2 Diffusion-weighted magnetic resonance imaging

Images were acquired at the National Laboratory for Magnetic Resonance Imaging (Lanirem) in Juriquilla, Queretaro, Mexico, using a 7 T Bruker Pharmascan preclinical MRI scanner and a 2×2 array head surface coil. The rats were anesthetized with isoflurane (4% for induction, 2% for maintenance) and kept warm by circulating water at 37 °C through hoses placed under the scanner bed. Vital signs were continuously monitored throughout the study using a compatible system. A single imaging session was performed for each animal (four months old) to determine diffusion parameters in the dysplastic and normal cortices. Each session lasted one hour. dMRI were obtained using an open-source sequence based on a 2D spin-echo echo-planar acquisition sequence, available from the Preclinical Neuro MRI repository (https://github.com/mdbudde/mcw_Preclinical_MRIsequences). Images with coronal orientation were acquired with voxel resolution of 200×200×1010 µm^3^, repetition time (TR) = 2000 ms, echo time (TE) = 40.86 ms, flip angle = 90°. The implemented protocol consisted of three b-tensor shapes: linear, spherical and planar [39]. The spherical tensor encoding (STE) gradients were numerically optimized and compensated for concomitant gradients prior to acquisition by using the NOW toolbox (https://github.com/jsjol/NOW) [40,41] tailored to minimize TE and scaled in magnitude to obtain four b-values (200, 700, 1400, and 2000 s/mm^2^). To retain gradient spectral characteristics between waveforms, the planar and linear tensor encoding gradients (LTE and PTE, respectively) were extracted from the STE waveform, using one axis for LTE and the other two for PTE [30]. The STE waveform was rotated in 10 directions at each b-value; LTE and PTE waveforms were rotated and scaled to obtain [10,16,46] directions for each corresponding b-value. S1 Fig shows the waveforms and protocol used in this study. The raw DWI are available at https://osf.io/n46d8.

### 2.3 DWI processing

**Fig 1:**
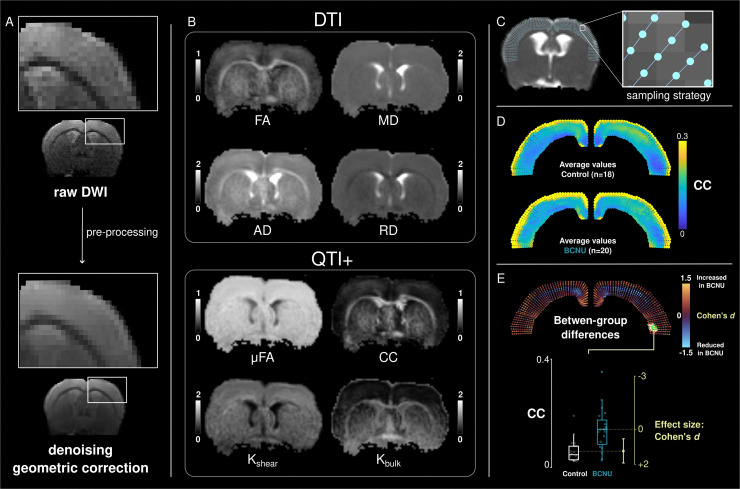
Analysis pipeline. A: Raw dMRI were preprocessed to minimize noise and correct for geometric inhomogeneities. B: QTI+ provided four DTI metrics (top): fractional anisotropy (FA), and axial, radial and mean diffusivities (AD, RD, MD, respectively; in units of mm^2^/s), as well as (bottom) microscopic anisotropy (µFA), microscopic orientation coherence (CC), and anisotropic and isotropic kurtosis (K_shear_ and K_bulk_, respectively). C: A curvilinear coordinate system (50 grid-lines spanning medial to lateral, and 10 depth levels from the pial border to the gray/white matter border) was defined in each rat brain, providing inter-subject anatomical correspondence. Descriptive statistics were obtained from the data sampled by the grid-lines, the point-wise average values are shown for each group (D). Student’s *t*-tests were subsequently performed at each point followed by correction for multiple comparisons (E). Marker size and color indicate effect size (Cohen’s *d*), gray circles indicate point-wise p_uncorr_ <0.01, white areas indicate significant clusters (p_clus_ <0.05). A Gardner-Altman plot presents data from one exemplary vertex (green circle in Cohen’s *d* map).

The images were preprocessed to minimize noise and artifacts. This included noise reduction [42] and correction of geometric inhomogeneities [43] using Mrtrix 3.0.4 [44] and fsl 6.0.7.1 [45] (Fig 1A). Q-space trajectory imaging with positivity constraints (QTI+) [46] was computed as implemented in https://github.com/DenebBoito/qtiplus. In addition to the four diffusion-tensor metrics, namely fractional anisotropy (FA), axial, radial and mean diffusivities (AD, RD and MD, respectively) (Fig 1B, top panel), QTI+ provides four complementary metrics: microscopic anisotropy (µFA), microscopic orientation coherence (CC), and mean anisotropic and isotropic kurtosis (K_shear_, and K_bulk_, respectively) [29] (Fig 1B, bottom panel). µFA quantifies the average anisotropy of compartments in a voxel disregarding their orientation, while CC is sensitive to the intra-voxel variability of their orientations. K_bulk_ informs of the heterogeneity in isotropic diffusivities (i.e., tensor sizes) across compartments, and is related to the variance of cell density, edema, and free water. K_shear_ informs of the variance in microscopic anisotropy and dispersion of microenvironments (i.e., tensor shapes and orientations), and helps separate tissue components by their geometry (e.g., axonal from non-axonal components). There is discrepancy in the literature related to the notation of some diffusion metrics [33]. In this work we use the notation described in [29]. These summary metrics simplify the interpretation of the distribution of tensors of different shapes, sizes, and orientations, and are sensitive to features of microarchitecture [28]. The valid range for FA, µFA and CC was between 0 and 1, and voxels outside of these ranges (less than 0.5% of all voxels) were attributed to fitting errors and excluded from further analyses.

### 2.4 Spatial analysis

A curvilinear coordinate system with anatomical correspondence between animals was constructed for each rat to sample dMRI parameters across the depth and extent of the cortex [27]. For this purpose, in a single coronal slice (located approximately −0.8 mm posterior to bregma, harboring primary motor and somatosensory cortices [M1 and S1, respectively]), the pial surface of the brain and the boundary between gray and white matter were manually delineated. A Laplacian potential field was simulated between these two boundaries [47]. From the pial surface, 50 evenly distributed virtual trajectories (grid-lines) were created and propagated organically through the Laplacian field; each trajectory extended towards the white matter boundary, following the curvature of the cortex in a manner analogous to cortical columns. The dMRI parameters were sampled at 10 equidistant points along each of these grid-lines (Fig 1C). The code to create this curvilinear grid is available at https://github.com/lconcha/Displasias.

### 2.5 Statistical analysis

Acknowledging that cortical cyto-and myeloarchitecture varies among cortical regions [48], the analysis was conducted in a spatially dependent fashion. Abnormalities were assessed separately for each dMRI metric using univariate statistics (Fig 1D, E). At each point in each grid-line, Student’s *t*-tests were performed to detect differences between the two groups (Fig 1E). Effect size estimation (the magnitude of the difference between groups) was calculated using Cohen’s *d*. Statistical analyses were corrected to minimize the probability of Type I errors (false positives) by using cluster-level permutation tests [49]. The vertex-wise cluster-forming threshold was set as p_uncorr_ < 0.01. Cluster significance (p_clus_) was determined by randomizing the data between experimental groups, performing 5,000 permutations to obtain the distribution of cluster sizes that could arise by chance. From this empirically-derived null distribution, the probability of finding clusters with a similar extent to those observed in the real data (i.e., without randomization between groups) was calculated. Cluster-wise statistical significance was defined as p_clus_ <0.05.

## 3 Histological analysis

After completing the dMRI studies all the animals were deeply anesthetized using an intraperitoneal overdose of sodium pentobarbital and intracardially perfused with 0.9% NaCl solution followed by 4% paraformaldehyde (PFA) solution. The brains were removed and preserved in fresh PFA 4% solution for 24 h at 4 °C. After this, each brain was immersed in a 20% sucrose solution for 48 h, followed by a 30% sucrose solution for another 48 h. Brains were stored at −72 °C until further analysis. Coronal sections (20 µm-thick) from the region of interest were obtained using a cryostat (Leica) based on the following Paxinos and Watson Rat Brain Atlas interaural coordinates: 8.74–8.08 mm. Slices were kept in a cold 1X phosphate buffer solution (PBS; Sigma-Aldrich). Immunofluorescence was performed using the primary antibodies anti-Myelin Basic Protein (MBP; 1:500; abcam), anti-Neuronal Nuclear Protein (NeuN; 1:350; abcam), and anti-Glial Fibrillary Acidic Protein (GFAP; 1:350; Sigma-Aldrich). For the triple immunofluorescence staining, the tissue sections were blocked with Bovine Serum Albumin (BSA; Sigma-Aldrich) 2% solution + 0.3% triton X-100 (ThermoFisher) in 1X PBS for 45 min. The sections were incubated with the primary antibodies (MBP and NeuN) for 24 h at 4°C. Then, slices were washed five times for 10 min in PBS 1X + Tween 0.1% solution (Sigma-Aldrich). Secondary antibodies conjugated with fluorescent dyes (AlexaFluor, goat anti-mouse-647 and goat anti-rabbit-555) were diluted 1:500 in a solution of PBS 1X + 0.1% Tween and incubated for 4 h at 4°C. After incubation, the slices underwent five washes (each one lasting 10 min) in PBS 1X. The sections were then blocked with a solution of BSA 2% + 0.3% Triton X-100 in PBS 1X for 45 min. Finally, GFAP was added in a PBS 1X + 0.1% Tween solution and incubated for 24 h at 4°C. Afterwards, another set of five 10-min washes in PBS 1X + 0.1% Tween was done, and the corresponding secondary antibody was added (AlexaFluor, goat anti-mouse-488) for 4 h, followed by five additional washes in 1X PBS. Finally, the slices were mounted using Mowiol. Brain slices were imaged using a confocal microscope (Zeiss LSM 880, with 488/594/647 nm wavelengths) and a fluorescence microscope (Zeiss Apotome, with 488/594 nm wavelengths). The system of this last microscope was linked to a computer running AxioVision software (version 4.8), where the MosaiX module was used to acquire mosaic images at 10X. Mosaics are available at https://osf.io/n46d8.

The photomicrographs were evaluated using Fiji [50]. Samples were taken from the primary motor cortex (M1) and the primary somatosensory cortex (S1). In these samples, the brightness threshold was automatically adjusted [51], and the spatial profile of glial density was also determined by calculating the percentage of the area occupied by the cells. The organization of the myeloarchitecture was evaluated through structure tensor analysis [52], as implemented in OrientationJ (https://github.com/Biomedical-Imaging-Group/OrientationJ) [53], calculating vector and local coherency maps using a Gaussian window of 15 µm. From these, we performed between-group comparisons of texture coherency and energy metrics, as well as principal texture orientation with respect to the pial boundary, using Student’s *t*-tests and computed profiles of said metrics as a function of cortical depth.

## 4 Results

### 4.1 Analysis of diffusion-weighted magnetic resonance images

**Fig 2:**
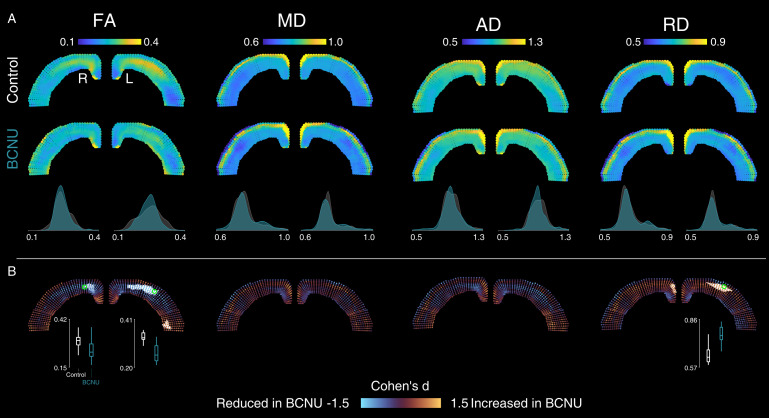
DTI metrics. A: Average values for each metric are shown for control and BCNU-treated animals (first two rows). Histograms show the group-wise average values across the entire cortex per hemisphere (third row). B: Between-group differences illustrated as in Fig 1E. Effect sizes (Cohen’s *d*) are color-coded at each point per grid line. White areas indicate cluster-corrected statistical significance (p_clus_ <0.05). Box plots of vertices identified in green are shown below hemispheres with significant clusters.

**Fig 3:**
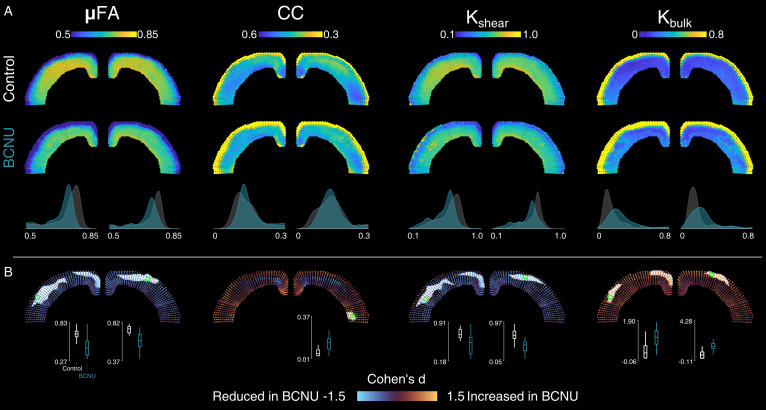
QTI+ metrics. Figure layout and description as in Fig 2.

All diffusion metrics showed spatial variability across the extent and depth of the cortex, which is not captured by histogram analyses of the entire cortex (Figs 2 and 3). In control animals, the middle layers of the cortex showed the highest FA and lowest RD in the middle layers of M1 and S1 regions. Contrarily, the most lateral aspects of the cortex showed the lowest average FA values. Mean and axial diffusivities were less heterogeneous across the cortex, with the exception of the most superficial layers. Metrics derived from QTI+ in control animals showed very high values of µFA in the middle layers of the cortex with decreasing values towards the lateral aspects of the cortex. This pattern was mirrored by K_shear_. A band of very high CC and K_bulk_ values was seen in the most superficial layers of the cortex. Average between-group differences are readily visible for µFA, K_shear_, and K_bulk_ in the spatial maps and in the histogram analyses.

BCNU-treated animals showed decreased FA values in the central-medial areas of the cortex in both hemispheres, encompassing the motor cortices M1 and M2, the primary somatosensory, and the cingulate cortex (Fig 2). This reduction of FA was accompanied by an increase of axial diffusivity and a reduction of axial diffusivity that was significant at the cluster level in the left hemisphere. Mean and axial diffusivities did not show any statistically significant differences between groups. QTI+ metrics attainable only through b-tensor encoding provided additional information (Fig 3). Both microscopic anisotropy and anisotropic kurtosis showed extended reductions along the cortex of both hemispheres in BCNU-treated animals (including cingulate, motor, and somatosensory cortex), mostly in the middle to superficial layers. The deep layers of the most lateral aspect of the left hemisphere showed an increased of CC, while the middle layers of the rest of the cortex showed reductions that were not significant at the cluster level. Finally, there were distributed increases of K_bulk_ throughout the superficial layers of the cortex in both hemispheres.

### 4.2 Histology

**Fig 4:**
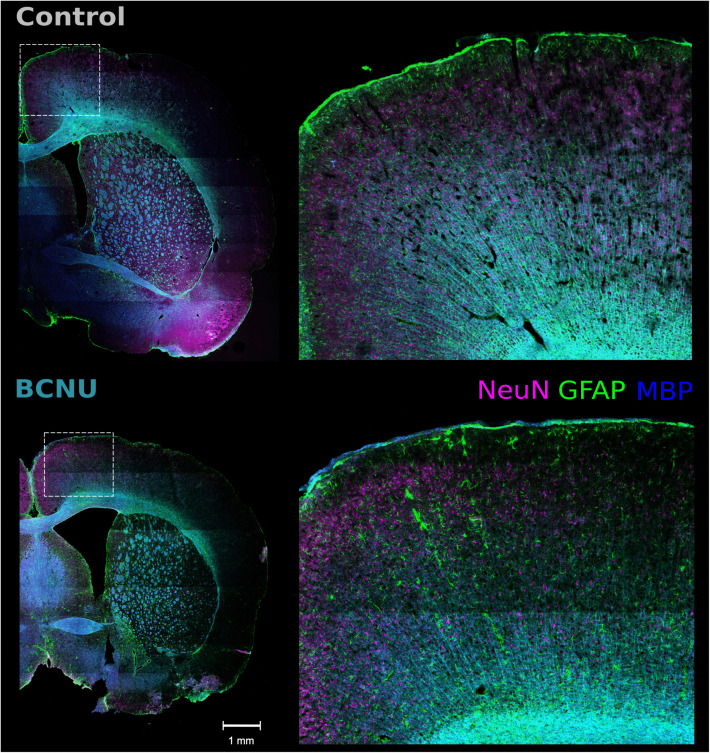
Triple-labelled Immunofluorescence showing neuronal nuclei (NeuN; in magenta, 594 nm), glia (GFAP; in green, 488 nm), and myelinated fibers (MBP; in blue, 647 nm) from a control (top) and a BCNU-treated rat (bottom). The experimental animal shows overall less intracortical myelin, heterogeneous spatial distribution of neuronal nuclei, and increased astrocytic processes.

Qualitative evaluations revealed reduced intracortical myelination in BCNU-treated rats, with reduced MBP+ fibers in the most superficial cortical layers. Additionally, variations in the distribution of neuronal nuclei (NeuN+) and morphological/quantitative changes in astrocytes were observed in BCNU-treated rats compared to the control group (Fig 4).

#### 4.2.1 Myelin analysis.

**Fig 5:**
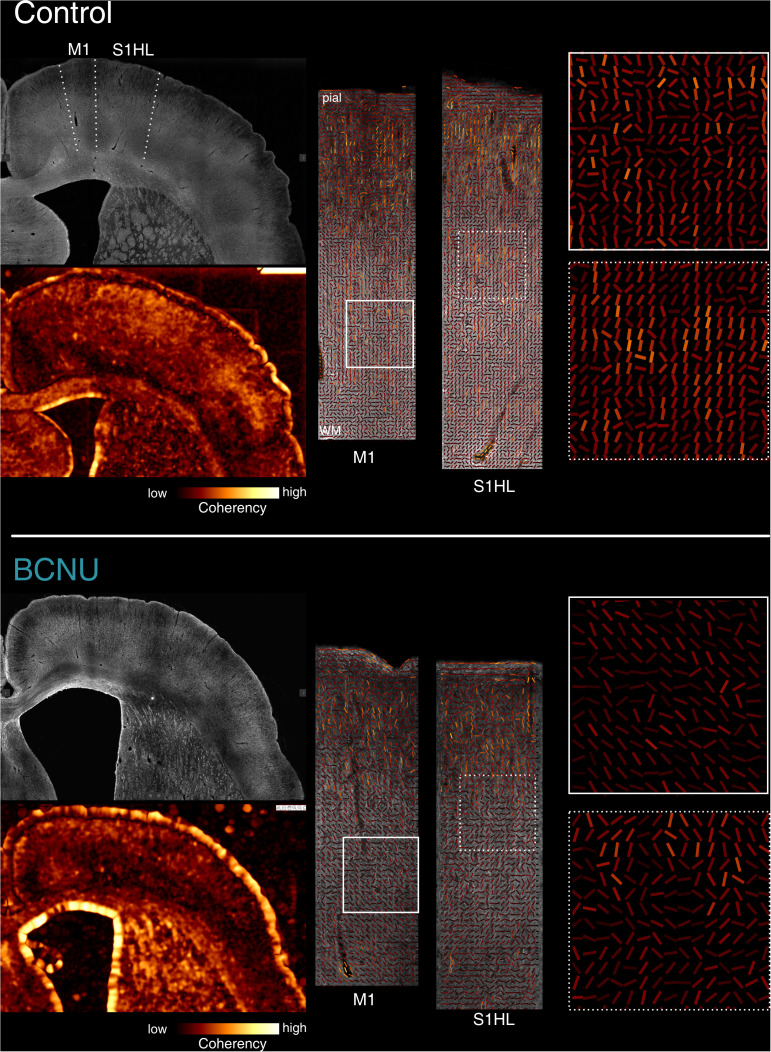
Structure tensor analysis of MBP+ fibers. Exemplary control (top) and BCNU-treated (bottom) animals showing MBP+ fibers (grayscale) and corresponding coherency maps (warm colors) derived from structure tensor analysis. For each animal, primary motor (M1) and primary somatosensory cortex (hindlimbs region, S1HL) are shown enlarged in the middle panels. Solid and dotted squares indicate the regions magnified in the rightmost panels. Experimental animals show reduced coherency throughout the cortex and magnifications illustrate coherent directions that are observed in the control group, while experimental animals show less coherency and interspersed vortices. Depth-wise analyses for M1 and S1HL are shown in S2 Fig.

Quantitative examination of histology through structure tensor analysis of MBP-labelled immunofluorescent photomicrographs revealed differences of the myeloarchitecture of the cerebral cortex between the control and experimental animals. BCNU-treated animals showed decreased coherence from the medial to the most lateral region of the cortex. This was further corroborated by vector maps, where interspersed vector vortices were observed in the somatosensory region, indicating a disorganization of myelin fibers (Figs 5 and S2).

#### 4.2.2 Histological analysis of astrocyte cortical distribution.

**Fig 6:**
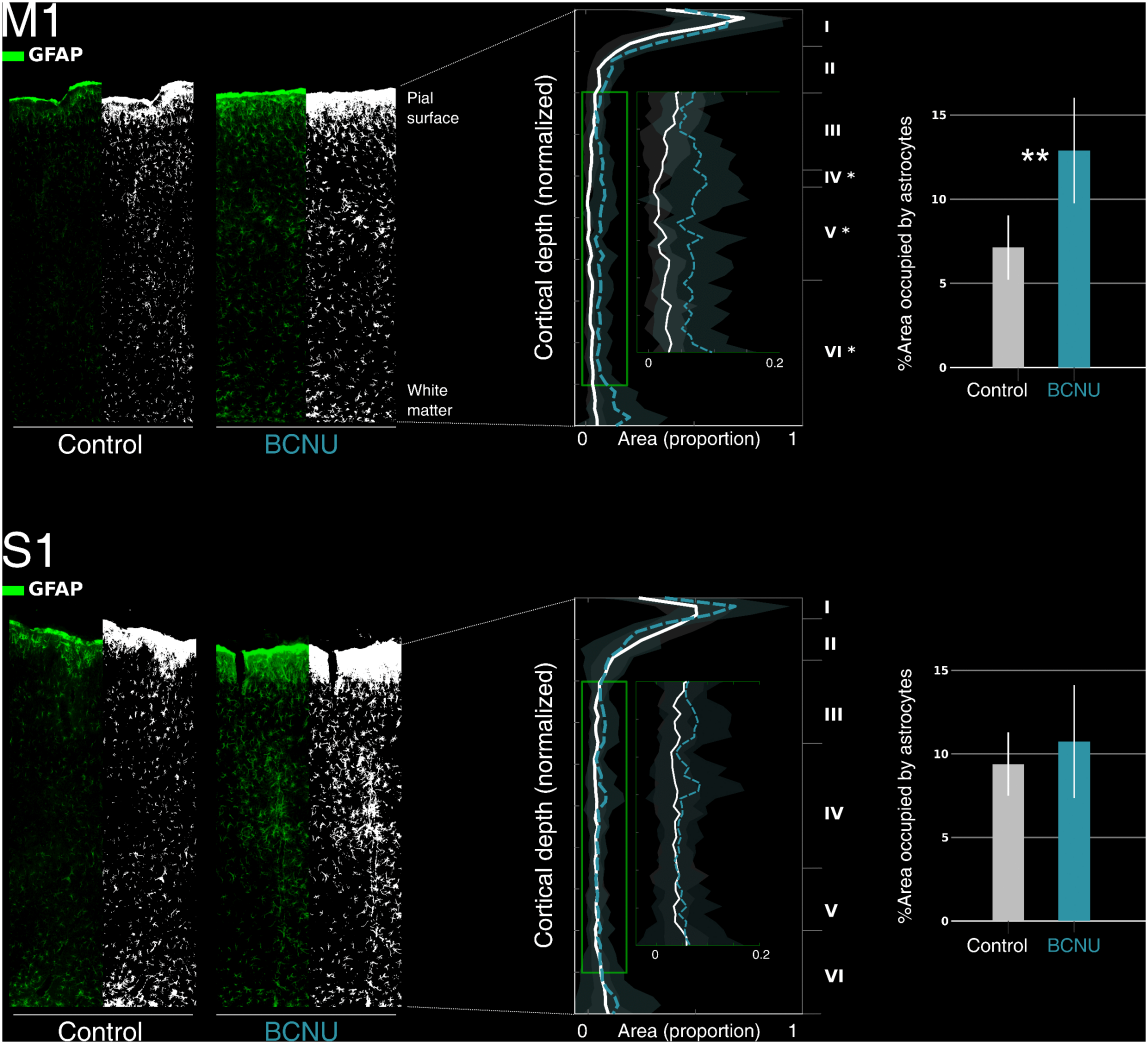
Quantification of area occupied by astrocytes (GFAP+) in the cortical regions M1 (top row) and S1 (hindlimb region, bottom row). For each cortical region, left-most panels show the GFAP immunofluorescence (green) in the control and BCNU groups, and the corresponding binarized image (black and white). Middle panels show the group-wise, depth-dependent spatial density profiles, with layers II-V enlarged in the insets. GFAP+ area is larger in experimental animals, particularly in layers IV-VI. Bar graphs in the rightmost panels show between group differences throughout the entire cortical region (**: p < 0.01).

There was an overall increase of the percentage of area occupied by astrocytes (GFAP+) between the control and experimental groups in the primary motor cortex (Fig 6, top row). Depth-wise analysis revealed increased GFAP+ area particularly in cortical layers IV-VI (Fig 6, middle panel). This increased presence of glial processes was not as marked in the S1 region (Fig 6, bottom row).

## 5 Discussion

Despite the high spatial resolution attainable with modern anatomical MRI, identification of FCD in patients with focal-onset epilepsy remains a challenge, as their microscopic disarray can hide behind tissue that looks deceptively ordinary at macroscopic scales. In this work we show that advanced dMRI with b-tensor encoding is able to extract information at the mesoscopic level, evidencing subtle histopathological landmarks characteristic of cortical malformations.

We used an animal model that presents the subtle histological cyto- and myeloarchitectonic irregularities characteristic of human FCD [27,34,37]. In prior work from our group using the same model we showed that multi-tensor fit of the diffusion signal was able to separate groups of intracortical fibers depending on their orientation to the cortical surface (i.e., radial and tangential fibers) and, moreover, differentially identify diffusion abnormalities in BCNU-treated animals [27]. Here, we used QTI+ to derive diffusion tensor metrics, and found reductions of FA in the middle cortical layers, in close agreement with our previous report [27], despite the age difference of the rats between the two studies (P30 vs P120). These results are in line with previous reports of reduced FA within FCD lesions [54,55] and also in the superficial white matter adjacent to the lesions [56–58]. DTI is a commonplace method, available in virtually all MRI scanners. The robustness of the FA findings across studies indicates that this metric has the potential to aid in the identification of FCD in patients [59]. Its sensitivity, however, is likely reduced as a consequence of the known limitations of DTI that render it insufficient to characterize the complex organization of the neocortex. Previous reports have shown the utility of diffusion metrics derived from neurite orientation dispersion and density imaging (NODDI) [60], such as microscopic anisotropy and intracellular and intra-neurite volume fractions, for the detection of FCD [57,61,62]. Notably, NODDI is specifically designed to characterize white matter, and therefore may be unfit for the study of the neocortex. Extending the ideas of NODDI, soma and neurite density imaging (SANDI) provides an approximation to the biophysical properties of gray matter with the inclusion of a sphere compartment to model cell bodies [63]. Metrics derived from SANDI are good indicators of gray matter microstructure in animal models [64], as well as in humans [65–67], and therefore constitute a viable option for the detection of FCD in future studies.

The single diffusion encoding method [20] is extremely useful for the acquisition of DWI and their subsequent analyses through many different methods. Nonetheless, acquisition schemes with free gradient waveforms sample the signal more richly across measurement space [29]. From these, complementary diffusion metrics tailored to separate microscopic anisotropy from orientation dispersion and other sources of variance can be obtained through the examination of diffusion tensor distributions [28,29,46]. Acquiring independent observations in a multidimensional manner enhances the characterization of heterogeneous media [30]. In our work, QTI+ metrics showed reductions of microscopic anisotropy that were more extensive than the corresponding DTI abnormalities, as evidenced by the two correlated metrics µFA and K_shear_. These reductions can be interpreted as compromised axonal structure [68–70]. In parallel, K_bulk_ showed an overall increase in the superficial layers of the cortex in BCNU animals, concordant with gliosis and heterogeneity of cellular size and morphology [71,72]. Our histological examinations, as well as prior evaluations of glial cells in BCNU-treated animals [73] support this interpretation. The utility of b-tensor encoding for the detection of different forms of malformations of cortical development has been explored by Lampinen *et al*. [74], with findings in FCD that are in line with our results. Said authors showed relatively low values of microscopic diffusion anisotropy in FCD lesions. Interestingly, regions of increased microscopic anisotropy were observed in the deepest portion of FCD lesions that correspond to the blurred gray/white matter boundary—which the authors interpret as myelin abnormalities of the superficial white matter. In rodents, the cortex is immediately adjacent to very large white matter fascicles (i.e., the corpus callosum and external capsules), contrasting with the relatively large volume of superficial white matter observed in humans [75]. This disposition of fibers makes analysis of juxtacortical white matter of our experimental animals difficult, but in humans the superficial white matter may provide information useful for the identification of cortical malformations [58]. The added value of b-tensor encoding and QTI + , together with the feasibility to perform this type of acquisition efficiently in the clinic [76] open new avenues for the detection of FCD.

The animal model used here shows histopathological features similar to those observed clinically [38], including cortical dyslamination and disarray of the myeloarchitecture [27,35,37]. Reductions in microscopic orientation coherence (CC) were observed in the middle cortex of BCNU-treated animals (Fig 3) and are likely associated with the altered geometric disposition of myelinated fibers (Figs 4 and 5), despite not reaching cluster-level significance. Of note, an increase in glial processes was observed in BCNU-treated animals compared to controls, especially in layers IV-VI of M1 region (Figs 4 and 6). Contrastingly, S1 showed no significant astrocytic density change. Differences of cortical architecture may render M1 more susceptible to microstructural disruption [77,78]. Prominent gliosis due to oxidative stress and inflammation is a common finding in human dysplastic cortex and in many non-genetic animal models [79–81]. While ongoing seizures are a cause of reactive gliosis, astrocytic gliosis alone is capable of initiating epileptic activity [82]. Identification of focal gliosis is, therefore, another opportunity for the detection of FCD, and has in fact been explored with, for example, radiotracers [83] and manganese-enhanced MRI [84]. In agreement with other reports that used dMRI to detect gliosis [85–87], our QTI+ results are indicative of this possibility.

Our study has limitations to consider. While the animal model used here induces alterations of cortical microarchitecture typical of FCD, it does not encompass the range of abnormalities seen in humans. Notably, the cortex of BCNU-treated animals lacks balloon cells, a prominent feature of FCD Type IIb; consequently, the model used more closely resembles FCD Type IIa [88]. Therefore, we cannot ascertain the impact that such abnormal cells may have on the diffusion signal, nor if milder alterations of cyto- and myeloarchitecture characteristic of FCD Type I are enough to alter diffusion metrics. Evaluation of the sensitivity and specificity of diffusion metrics to detect FCD would require a gold standard to be compared to. However, the cortical abnormalities induced by BCNU are not focal but rather distributed across the cortex, which contrasts to the moderately well-demarcated FCD lesions observed in humans. Other animal models, such as freeze lesions of the cortex, induce focal alterations [89], but can include macroscopic abnormalities and the creation of a microgyrus [90]. Such gross anatomical alterations are easily identified with conventional MRI, thus rendering dMRI unnecessary, and are against our ultimate goal to detect the subtle mesoscopic abnormalities that often go unnoticed in patients. While all animals were processed for histology, technical difficulties during tissue handling preclude a one-to-one comparison of dMRI and microphotographs, and the evaluation of correlations between diffusion metrics and quantitative histology. Diffusion time dependence and water exchange effects, which may modulate QTI+ and other dMRI metrics [91], were not accounted for in this study. This effect may be even more relevant in gray matter given its abundance of non-myelinated (and therefore more water-permeable) axons and dendrites [92–94]. In this study we took the common approach for tuning the waveforms by taking one axis of the STE waveform to construct the LTE waveform, and the other two perpendicular axes to construct the PTE waveform. However, recent studies have shown that the metrics could be biased in the presence of time-dependence effects [95,96]. Future studies should further optimize their acquisition [95] or fitting procedures [96] to properly account for these effects. We attempted to optimize the diffusion gradient waveforms using the NOW toolbox [40] while aiming to minimize echo time as much as possible, which led to waveforms that are somewhat suboptimal. While this is unlikely to impact on the presented results, we warn readers that the diffusion gradient waveforms we present in S1 Fig may be further optimized. Finally, although b-tensor encoding is feasible in clinical scanners [76], spatial resolution will be an important hurdle to overcome in order to provide sufficient sampling of the human cortical mantle, which typically has a thickness of 1.5–4.5 mm [97], thus making super-resolution techniques highly desirable [98].

Our imaging and histology findings paint a coherent picture that highlights the ability of b-tensor encoding to capture the subtle histopathological abnormalities present in FCD. QTI+ resolved fine-grained attributes (microstructural anisotropy loss, orientation dispersion, and intra-voxel heterogeneity) closely tracking myelin disorganization and glial changes. The benefits of b-tensor encoding and QTI + are especially valuable in the cortex, where the complex architecture of the tissue poses challenges to simpler diffusion models.

## Supporting information

S1 Fig Diffusion gradient waveforms. Three exemplary waveforms are shown for spherical, planar, and linear tensor encodings (STE, PTE and LTE, respectively). STE waveforms were used as a base to create PTE waveforms from its Gx and Gy components, and LTE waveforms using Gz. Gradient amplitude (mT/m) was scaled to create different b value scalings. The table at the top right shows the number of directions for each b-tensor shape and b value scaling. Further optimization of these gradient waveforms is possible and should be considered in future studies.
(TIF)

S2 Fig Group comparisons of texture analyses of immunofluorescence microphotographs of myelin basic protein (MBP) in primary motor cortex (M1) and primary somatosensory cortex (hindlimbs region, S1HL). Depth-wise averages of coherency, energy and radiality (calculated as the absolute dot product between the main orientation of MBP and the normal vector of the pial surface) are shown as mean (lines) ± 1 standard deviation (shared areas). Insets show boxplots for the average values across the entire region, with p-values for individual t-tests.
(TIF)

## References

[1] Blumcke I, Spreafico R, Haaker G, Coras R, Kobow K, Bien CG, et al. Histopathological Findings in Brain Tissue Obtained during Epilepsy Surgery. N Engl J Med. 2017;377(17):1648–56. doi: 10.1056/NEJMoa1703784 29069555

[2] Guerrini R, Barba C. Focal cortical dysplasia: an update on diagnosis and treatment. Expert Rev Neurother. 2021;21(11):1213–24. doi: 10.1080/14737175.2021.1915135 33834938

[3] Bernasconi A, Bernasconi N, Bernhardt BC, Schrader D. Advances in MRI for “cryptogenic” epilepsies. Nat Rev Neurol. 2011;7(2):99–108. doi: 10.1038/nrneurol.2010.199 21243016

[4] Walger L, Schmitz MH, Bauer T, Kügler D, Schuch F, Arendt C, et al. A public benchmark for human performance in the detection of focal cortical dysplasia. Epilepsia Open. 2025;10(3):778–86. doi: 10.1002/epi4.70028 40167314 PMC12163524

[5] Colombo N, Salamon N, Raybaud C, Ozkara C, Barkovich AJ. Imaging of malformations of cortical development. Epileptic Disord. 2009;11(3):194–205. doi: 10.1684/epd.2009.0262 19720583

[6] Blackmon K, Kuzniecky R, Barr WB, Snuderl M, Doyle W, Devinsky O, et al. Cortical Gray-White Matter Blurring and Cognitive Morbidity in Focal Cortical Dysplasia. Cereb Cortex. 2015;25(9):2854–62. doi: 10.1093/cercor/bhu080 24770710

[7] Lee JW. Frontal Focal Cortical Dysplasias: Too Thin Here, Too Thick There, and the Folding Just Isn’t Right!. Epilepsy Curr. 2016;16(4):247–8. doi: 10.5698/1535-7511-16.4.247 27582663 PMC4988081

[8] Bernasconi A, Antel SB, Collins DL, Bernasconi N, Olivier A, Dubeau F, et al. Texture analysis and morphological processing of magnetic resonance imaging assist detection of focal cortical dysplasia in extra-temporal partial epilepsy. Ann Neurol. 2001;49(6):770–5. doi: 10.1002/ana.1013.abs 11409429

[9] Colliot O, Bernasconi N, Khalili N, Antel SB, Naessens V, Bernasconi A. Individual voxel-based analysis of gray matter in focal cortical dysplasia. Neuroimage. 2006;29(1):162–71. doi: 10.1016/j.neuroimage.2005.07.021 16099679

[10] Gill RS, Lee H-M, Caldairou B, Hong S-J, Barba C, Deleo F, et al. Multicenter Validation of a Deep Learning Detection Algorithm for Focal Cortical Dysplasia. Neurology. 2021;97(16):e1571–82. doi: 10.1212/WNL.0000000000012698 34521691 PMC8548962

[11] Spitzer H, Ripart M, Whitaker K, D’Arco F, Mankad K, Chen AA, et al. Interpretable surface-based detection of focal cortical dysplasias: a Multi-centre Epilepsy Lesion Detection study. Brain. 2022;145(11):3859–71. doi: 10.1093/brain/awac224 35953082 PMC9679165

[12] Su T-Y, Choi JY, Hu S, Wang X, Blümcke I, Chiprean K, et al. Multiparametric Characterization of Focal Cortical Dysplasia Using 3D MR Fingerprinting. Ann Neurol. 2024;96(5):944–57. doi: 10.1002/ana.27049 39096056 PMC11496021

[13] Ding Z, Morris S, Hu S, Su TY, Choi JY, Blümcke I. Automated whole-brain focal cortical dysplasia detection using MR fingerprinting with deep learning. Neurology. 2025;104(11):e213691. doi: 10.1212/wnl.0000000000213691 PMC12089660 40378378

[14] Beaulieu C. The basis of anisotropic water diffusion in the nervous system - a technical review. NMR Biomed. 2002;15(7–8):435–55. doi: 10.1002/nbm.782 12489094

[15] Concha L. A macroscopic view of microstructure: using diffusion-weighted images to infer damage, repair, and plasticity of white matter. Neuroscience. 2014;276:14–28. doi: 10.1016/j.neuroscience.2013.09.004 24051366

[16] Ghaderi S, Mohammadi S, Fatehi F. A systematic review of diffusion microstructure imaging (DMI): Current and future applications in neurology research. Brain Disorders. 2025;19:100238. doi: 10.1016/j.dscb.2025.100238

[17] Fountain C, Ghuman H, Paldino M, Tamber M, Panigrahy A, Modo M. Acquisition and Analysis of Excised Neocortex from Pediatric Patients with Focal Cortical Dysplasia Using Mesoscale Diffusion MRI. Diagnostics (Basel). 2023;13(9):1529. doi: 10.3390/diagnostics13091529 37174921 PMC10177920

[18] Alexander DC, Dyrby TB, Nilsson M, Zhang H. Imaging brain microstructure with diffusion MRI: practicality and applications. NMR Biomed. 2017. doi: 10.1002/nbm.3841 29193413

[19] Zhu A, Michael ES, Li H, Sprenger T, Hua Y, Lee S-K, et al. Engineering clinical translation of OGSE diffusion MRI. Magn Reson Med. 2025;94(3):913–36. doi: 10.1002/mrm.30510 40331336 PMC12262058

[20] Stejskal EO, Tanner JE. Spin Diffusion Measurements: Spin Echoes in the Presence of a Time-Dependent Field Gradient. J Chem Phys. 1965;42(1):288–92. doi: 10.1063/1.1695690

[21] Aggarwal M, Nauen DW, Troncoso JC, Mori S. Probing region-specific microstructure of human cortical areas using high angular and spatial resolution diffusion MRI. Neuroimage. 2015;105:198–207. doi: 10.1016/j.neuroimage.2014.10.053 25449747 PMC4262592

[22] Feizollah S, Tardif CL. 3D MERMAID: 3D Multi-shot enhanced recovery motion artifact insensitive diffusion for submillimeter, multi-shell, and SNR-efficient diffusion imaging. Magn Reson Med. 2025;93(6):2311–30. doi: 10.1002/mrm.30436 40035173 PMC11971498

[23] Leuze CWU, Anwander A, Bazin P-L, Dhital B, Stüber C, Reimann K, et al. Layer-specific intracortical connectivity revealed with diffusion MRI. Cereb Cortex. 2014;24(2):328–39. doi: 10.1093/cercor/bhs311 23099298 PMC3888365

[24] McNab JA, Polimeni JR, Wang R, Augustinack JC, Fujimoto K, Stevens A, et al. Surface based analysis of diffusion orientation for identifying architectonic domains in the in vivo human cortex. Neuroimage. 2013;69:87–100. doi: 10.1016/j.neuroimage.2012.11.065 23247190 PMC3557597

[25] Reveley C, Ye FQ, Leopold DA. Diffusion kurtosis imaging, MAP-MRI and NODDI selectively track gray matter myelin density in the primate cerebral cortex. Imaging Neurosci (Camb). 2024;2:imag–2–00368. doi: 10.1162/imag_a_00368 40800513 PMC12315753

[26] Reveley C, Ye FQ, Mars RB, Matrov D, Chudasama Y, Leopold DA. Diffusion MRI anisotropy in the cerebral cortex is determined by unmyelinated tissue features. Nat Commun. 2022;13(1):6702. doi: 10.1038/s41467-022-34328-z 36335105 PMC9637141

[27] Villaseñor PJ, Cortés-Servín D, Pérez-Moriel A, Aquiles A, Luna-Munguía H, Ramirez-Manzanares A, et al. Multi-tensor diffusion abnormalities of gray matter in an animal model of cortical dysplasia. Front Neurol. 2023;14:1124282. doi: 10.3389/fneur.2023.1124282 37342776 PMC10278582

[28] Topgaard D. Diffusion tensor distribution imaging. NMR Biomed. 2019;32(5):e4066. doi: 10.1002/nbm.4066 30730586 PMC6593682

[29] Westin C-F, Knutsson H, Pasternak O, Szczepankiewicz F, Özarslan E, van Westen D, et al. Q-space trajectory imaging for multidimensional diffusion MRI of the human brain. Neuroimage. 2016;135:345–62. doi: 10.1016/j.neuroimage.2016.02.039 26923372 PMC4916005

[30] Lundell H, Nilsson M, Dyrby TB, Parker GJM, Cristinacce PLH, Zhou F-L, et al. Multidimensional diffusion MRI with spectrally modulated gradients reveals unprecedented microstructural detail. Sci Rep. 2019;9(1):9026. doi: 10.1038/s41598-019-45235-7 31227745 PMC6588609

[31] Szczepankiewicz F, Westin C-F, Nilsson M. Gradient waveform design for tensor-valued encoding in diffusion MRI. J Neurosci Methods. 2021;348:109007. doi: 10.1016/j.jneumeth.2020.109007 33242529 PMC8443151

[32] Magdoom KN, Pajevic S, Dario G, Basser PJ. A new framework for MR diffusion tensor distribution. Sci Rep. 2021;11(1):2766. doi: 10.1038/s41598-021-81264-x 33531530 PMC7854653

[33] Syed Nasser N, Rajan S, Venugopal VK, Lasič S, Mahajan V, Mahajan H. A review on investigation of the basic contrast mechanism underlying multidimensional diffusion MRI in assessment of neurological disorders. J Clin Neurosci. 2022;102:26–35. doi: 10.1016/j.jocn.2022.05.027 35696817

[34] Benardete EA, Kriegstein AR. Increased excitability and decreased sensitivity to GABA in an animal model of dysplastic cortex. Epilepsia. 2002;43(9):970–82. doi: 10.1046/j.1528-1157.2002.40901.x 12199722

[35] Inverardi F, Chikhladze M, Donzelli A, Moroni RF, Regondi MC, Pennacchio P, et al. Cytoarchitectural, behavioural and neurophysiological dysfunctions in the BCNU-treated rat model of cortical dysplasia. Eur J Neurosci. 2013;37(1):150–62. doi: 10.1111/ejn.12032 23095101

[36] Moroni RF, Inverardi F, Regondi MC, Panzica F, Spreafico R, Frassoni C. Altered spatial distribution of PV-cortical cells and dysmorphic neurons in the somatosensory cortex of BCNU-treated rat model of cortical dysplasia. Epilepsia. 2008;49(5):872–87. doi: 10.1111/j.1528-1167.2007.01440.x 18076647

[37] Aquiles A, Fiordelisio T, Luna-Munguia H, Concha L. Altered functional connectivity and network excitability in a model of cortical dysplasia. Sci Rep. 2023;13(1):12335. doi: 10.1038/s41598-023-38717-2 37518675 PMC10387479

[38] Najm I, Lal D, Alonso Vanegas M, Cendes F, Lopes-Cendes I, Palmini A, et al. The ILAE consensus classification of focal cortical dysplasia: An update proposed by an ad hoc task force of the ILAE diagnostic methods commission. Epilepsia. 2022;63(8):1899–919. doi: 10.1111/epi.17301 35706131 PMC9545778

[39] Szczepankiewicz F, Hoge S, Westin C-F. Linear, planar and spherical tensor-valued diffusion MRI data by free waveform encoding in healthy brain, water, oil and liquid crystals. Data Brief. 2019;25:104208. doi: 10.1016/j.dib.2019.104208 31338402 PMC6626882

[40] Sjölund J, Szczepankiewicz F, Nilsson M, Topgaard D, Westin C-F, Knutsson H. Constrained optimization of gradient waveforms for generalized diffusion encoding. J Magn Reson. 2015;261:157–68. doi: 10.1016/j.jmr.2015.10.012 26583528 PMC4752208

[41] Szczepankiewicz F, Westin C-F, Nilsson M. Maxwell-compensated design of asymmetric gradient waveforms for tensor-valued diffusion encoding. Magn Reson Med. 2019;82(4):1424–37. doi: 10.1002/mrm.27828 31148245 PMC6626569

[42] Cordero-Grande L, Christiaens D, Hutter J, Price AN, Hajnal JV. Complex diffusion-weighted image estimation via matrix recovery under general noise models. Neuroimage. 2019;200:391–404. doi: 10.1016/j.neuroimage.2019.06.039 31226495 PMC6711461

[43] Andersson JLR, Sotiropoulos SN. An integrated approach to correction for off-resonance effects and subject movement in diffusion MR imaging. Neuroimage. 2016;125:1063–78. doi: 10.1016/j.neuroimage.2015.10.019 26481672 PMC4692656

[44] Tournier J-D, Smith R, Raffelt D, Tabbara R, Dhollander T, Pietsch M, et al. MRtrix3: A fast, flexible and open software framework for medical image processing and visualisation. Neuroimage. 2019;202:116137. doi: 10.1016/j.neuroimage.2019.116137 31473352

[45] Smith SM, Jenkinson M, Woolrich MW, Beckmann CF, Behrens TEJ, Johansen-Berg H, et al. Advances in functional and structural MR image analysis and implementation as FSL. Neuroimage. 2004;23 Suppl 1:S208–19. doi: 10.1016/j.neuroimage.2004.07.051 15501092

[46] Herberthson M, Boito D, Haije TD, Feragen A, Westin C-F, Özarslan E. Q-space trajectory imaging with positivity constraints (QTI+). NeuroImage. 2021;238:118198. doi: 10.1016/j.neuroimage.2021.118198 34029738 PMC9596133

[47] Lerch JP, Pruessner J, Zijdenbos AP, Collins DL, Teipel SJ, Hampel H, et al. Automated cortical thickness measurements from MRI can accurately separate Alzheimer’s patients from normal elderly controls. Neurobiol Aging. 2008;29(1):23–30. doi: 10.1016/j.neurobiolaging.2006.09.013 17097767

[48] Kleven H, Bjerke IE, Clascá F, Groenewegen HJ, Bjaalie JG, Leergaard TB. Waxholm Space atlas of the rat brain: a 3D atlas supporting data analysis and integration. Nat Methods. 2023;20(11):1822–9. doi: 10.1038/s41592-023-02034-3 37783883 PMC10630136

[49] Cox RW, Chen G, Glen DR, Reynolds RC, Taylor PA. fMRI clustering and false-positive rates. Proc Natl Acad Sci U S A. 2017;114(17):E3370–1. doi: 10.1073/pnas.1614961114 28420798 PMC5410825

[50] Schindelin J, Arganda-Carreras I, Frise E, Kaynig V, Longair M, Pietzsch T, et al. Fiji: an open-source platform for biological-image analysis. Nat Methods. 2012;9(7):676–82. doi: 10.1038/nmeth.2019 22743772 PMC3855844

[51] Li CH, Tam PKS. An iterative algorithm for minimum cross entropy thresholding. Pattern Recog Lett. 1998;19(8):771–6. doi: 10.1016/s0167-8655(98)00057-9

[52] Budde MD, Frank JA. Examining brain microstructure using structure tensor analysis of histological sections. Neuroimage. 2012;63(1):1–10. doi: 10.1016/j.neuroimage.2012.06.042 22759994

[53] Püspöki Z, Storath M, Sage D, Unser M. Transforms and Operators for Directional Bioimage Analysis: A Survey. Adv Anat Embryol Cell Biol. 2016;219:69–93. doi: 10.1007/978-3-319-28549-8_3 27207363

[54] Gennari AG, Cserpan D, Stefanos-Yakoub I, Kottke R, O’Gorman Tuura R, Ramantani G. Diffusion tensor imaging discriminates focal cortical dysplasia from normal brain parenchyma and differentiates between focal cortical dysplasia types. Insights Imaging. 2023;14(1):36. doi: 10.1186/s13244-023-01368-y 36826756 PMC9958211

[55] Gross DW, Bastos A, Beaulieu C. Diffusion tensor imaging abnormalities in focal cortical dysplasia. Can J Neurol Sci. 2005;32(4):477–82. doi: 10.1017/s0317167100004479 16408578

[56] Lee S-K, Kim DI, Mori S, Kim J, Kim HD, Heo K, et al. Diffusion tensor MRI visualizes decreased subcortical fiber connectivity in focal cortical dysplasia. Neuroimage. 2004;22(4):1826–9. doi: 10.1016/j.neuroimage.2004.04.028 15275939

[57] Lorio S, Adler S, Gunny R, D’Arco F, Kaden E, Wagstyl K, et al. MRI profiling of focal cortical dysplasia using multi-compartment diffusion models. Epilepsia. 2020;61(3):433–44. doi: 10.1111/epi.16451 32065673 PMC7154549

[58] Urquia-Osorio H, Pimentel-Silva LR, Rezende TJR, Almendares-Bonilla E, Yasuda CL, Concha L, et al. Superficial and deep white matter diffusion abnormalities in focal epilepsies. Epilepsia. 2022;63(9):2312–24. doi: 10.1111/epi.17333 35707885

[59] Madan N, Grant PE. New directions in clinical imaging of cortical dysplasias. Epilepsia. 2009;50 Suppl 9:9–18. doi: 10.1111/j.1528-1167.2009.02292.x 19761449

[60] Zhang H, Schneider T, Wheeler-Kingshott CA, Alexander DC. NODDI: practical in vivo neurite orientation dispersion and density imaging of the human brain. Neuroimage. 2012;61(4):1000–16. doi: 10.1016/j.neuroimage.2012.03.072 22484410

[61] Rostampour M, Hashemi H, Najibi SM, Oghabian MA. Detection of structural abnormalities of cortical and subcortical gray matter in patients with MRI-negative refractory epilepsy using neurite orientation dispersion and density imaging. Phys Med. 2018;48:47–54. doi: 10.1016/j.ejmp.2018.03.005 29728228

[62] Winston GP, Micallef C, Symms MR, Alexander DC, Duncan JS, Zhang H. Advanced diffusion imaging sequences could aid assessing patients with focal cortical dysplasia and epilepsy. Epilepsy Res. 2014;108(2):336–9. doi: 10.1016/j.eplepsyres.2013.11.004 24315018 PMC3969285

[63] Palombo M, Ianus A, Guerreri M, Nunes D, Alexander DC, Shemesh N, et al. SANDI: A compartment-based model for non-invasive apparent soma and neurite imaging by diffusion MRI. Neuroimage. 2020;215:116835. doi: 10.1016/j.neuroimage.2020.116835 32289460 PMC8543044

[64] Ianuş A, Carvalho J, Fernandes FF, Cruz R, Chavarrias C, Palombo M, et al. Soma and Neurite Density MRI (SANDI) of the in-vivo mouse brain and comparison with the Allen Brain Atlas. Neuroimage. 2022;254:119135. doi: 10.1016/j.neuroimage.2022.119135 35339686

[65] Barakovic M, Weigel M, Cagol A, Schaedelin S, Galbusera R, Lu P-J, et al. A novel imaging marker of cortical “cellularity” in multiple sclerosis patients. Sci Rep. 2024;14(1):9848. doi: 10.1038/s41598-024-60497-6 38684744 PMC11059177

[66] Genc S, Ball G, Chamberland M, Raven EP, Tax CMW, Ward I, et al. MRI signatures of cortical microstructure in human development align with oligodendrocyte cell-type expression. Nat Commun. 2025;16(1):3317. doi: 10.1038/s41467-025-58604-w 40195348 PMC11977195

[67] Lee H, Lee H-H, Ma Y, Eskandarian L, Gaudet K, Tian Q, et al. Age-related alterations in human cortical microstructure across the lifespan: Insights from high-gradient diffusion MRI. Aging Cell. 2024;23(11):e14267. doi: 10.1111/acel.14267 39118344 PMC11561659

[68] Hansen B. An Introduction to Kurtosis Fractional Anisotropy. AJNR Am J Neuroradiol. 2019;40(10):1638–41. doi: 10.3174/ajnr.A6235 31558496 PMC7028548

[69] Li S, Zheng Y, Sun W, Lasič S, Szczepankiewicz F, Wei Q, et al. Glioma grading, molecular feature classification, and microstructural characterization using MR diffusional variance decomposition (DIVIDE) imaging. Eur Radiol. 2021;31(11):8197–207. doi: 10.1007/s00330-021-07959-x 33914116

[70] Shemesh N. Axon diameters and myelin content modulate microscopic fractional anisotropy at short diffusion times in fixed rat spinal cord. Front Phys. 2018. 10.3389/fphy.2018.00049

[71] Glenn GR, Helpern JA, Tabesh A, Jensen JH. Quantitative assessment of diffusional kurtosis anisotropy. NMR Biomed. 2015;28(4):448–59. doi: 10.1002/nbm.3271 25728763 PMC4378654

[72] Hui ES, Russell Glenn G, Helpern JA, Jensen JH. Kurtosis analysis of neural diffusion organization. Neuroimage. 2015;106:391–403. doi: 10.1016/j.neuroimage.2014.11.015 25463453 PMC4389769

[73] Rodríguez-Arzate CA, Martínez-Mendoza ML, Rocha-Mendoza I, Luna-Palacios Y, Licea-Rodríguez J, Martínez-Torres A. Morphological and Calcium Signaling Alterations of Neuroglial Cells in Cerebellar Cortical Dysplasia Induced by Carmustine. Cells. 2021;10(7):1581. doi: 10.3390/cells10071581 34201497 PMC8304447

[74] Lampinen B, Zampeli A, Björkman-Burtscher IM, Szczepankiewicz F, Källén K, Compagno Strandberg M, et al. Tensor-valued diffusion MRI differentiates cortex and white matter in malformations of cortical development associated with epilepsy. Epilepsia. 2020;61(8):1701–13. doi: 10.1111/epi.16605 32667688 PMC7963222

[75] Schilling KG, Archer D, Rheault F, Lyu I, Huo Y, Cai LY, et al. Superficial white matter across development, young adulthood, and aging: volume, thickness, and relationship with cortical features. Brain Struct Funct. 2023;228(3–4):1019–31. doi: 10.1007/s00429-023-02642-x 37074446 PMC10320929

[76] Nilsson M, Szczepankiewicz F, Brabec J, Taylor M, Westin C-F, Golby A, et al. Tensor-valued diffusion MRI in under 3 minutes: an initial survey of microscopic anisotropy and tissue heterogeneity in intracranial tumors. Magn Reson Med. 2020;83(2):608–20. doi: 10.1002/mrm.27959 31517401 PMC6900060

[77] Benedetti B, Dannehl D, Janssen JM, Corcelli C, Couillard-Després S, Engelhardt M. Structural and Functional Maturation of Rat Primary Motor Cortex Layer V Neurons. Int J Mol Sci. 2020;21(17):6101. doi: 10.3390/ijms21176101 32847128 PMC7503395

[78] Lee C, Kim Y, Kaang B-K. The Primary Motor Cortex: The Hub of Motor Learning in Rodents. Neuroscience. 2022;485:163–70. doi: 10.1016/j.neuroscience.2022.01.009 35051529

[79] Arena A, Zimmer TS, van Scheppingen J, Korotkov A, Anink JJ, Mühlebner A, et al. Oxidative stress and inflammation in a spectrum of epileptogenic cortical malformations: molecular insights into their interdependence. Brain Pathol. 2019;29(3):351–65. doi: 10.1111/bpa.12661 30303592 PMC8028690

[80] Kakita A, Kameyama S, Hayashi S, Masuda H, Takahashi H. Pathologic features of dysplasia and accompanying alterations observed in surgical specimens from patients with intractable epilepsy. J Child Neurol. 2005;20(4):341–50. doi: 10.1177/08830738050200041301 15921237

[81] Kielbinski M, Gzielo K, Soltys Z. Review: Roles for astrocytes in epilepsy: insights from malformations of cortical development. Neuropathol Appl Neurobiol. 2016;42(7):593–606. doi: 10.1111/nan.12331 27257021

[82] Robel S, Buckingham SC, Boni JL, Campbell SL, Danbolt NC, Riedemann T, et al. Reactive astrogliosis causes the development of spontaneous seizures. J Neurosci. 2015;35(8):3330–45. doi: 10.1523/JNEUROSCI.1574-14.2015 25716834 PMC4339349

[83] Butler T, Ichise M, Teich AF, Gerard E, Osborne J, French J, et al. Imaging inflammation in a patient with epilepsy due to focal cortical dysplasia. J Neuroimaging. 2013;23(1):129–31. doi: 10.1111/j.1552-6569.2010.00572.x 21223436 PMC5303618

[84] Kawai Y, Aoki I, Umeda M, Higuchi T, Kershaw J, Higuchi M, et al. In vivo visualization of reactive gliosis using manganese-enhanced magnetic resonance imaging. Neuroimage. 2010;49(4):3122–31. doi: 10.1016/j.neuroimage.2009.11.005 19909819 PMC5575780

[85] Benjamini D, Priemer DS, Perl DP, Brody DL, Basser PJ. Mapping astrogliosis in the individual human brain using multidimensional MRI. Brain. 2023;146(3):1212–26. doi: 10.1093/brain/awac298 35953450 PMC9976979

[86] Chary K, Manninen E, Claessens J, Ramirez-Manzanares A, Gröhn O, Sierra A. Diffusion MRI approaches for investigating microstructural complexity in a rat model of traumatic brain injury. Sci Rep. 2023;13(1):2219. doi: 10.1038/s41598-023-29010-3 36755032 PMC9908904

[87] Garcia-Hernandez R, Cerdán Cerdá A, Trouve Carpena A, Drakesmith M, Koller K, Jones DK, et al. Mapping microglia and astrocyte activation in vivo using diffusion MRI. Sci Adv. 2022;8(21):eabq2923. doi: 10.1126/sciadv.abq2923 35622913 PMC9140964

[88] Blümcke I, Coras R, Busch RM, Morita-Sherman M, Lal D, Prayson R, et al. Toward a better definition of focal cortical dysplasia: An iterative histopathological and genetic agreement trial. Epilepsia. 2021;62(6):1416–28. doi: 10.1111/epi.16899 33949696

[89] Lau LA, Dulla CG. Dysplasias: Cortical Freeze Lesion. In: Pitkänen A, Buckmaster PS, Galanopoulou AS, Moshé SL, editors. Models of Seizures and Epilepsy. 2nd ed. Academic Press; 2017. p. 845–59.

[90] Wong M. Animal models of focal cortical dysplasia and tuberous sclerosis complex: recent progress toward clinical applications. Epilepsia. 2009;50 Suppl 9(0 9):34–44. doi: 10.1111/j.1528-1167.2009.02295.x 19761452 PMC3934654

[91] Novikov DS, Jespersen SN, Kiselev VG, Fieremans E. Quantifying brain microstructure with diffusion MRI: Theory and parameter estimation. arXiv:161202059 [physics]. 2016. 10.1002/nbm.3998 PMC6481929 30321478

[92] Dong T, Lee H-H, Zang H, Lee H, Tian Q, Wan L, et al. In vivo cortical microstructure mapping using high-gradient diffusion MRI accounting for intercompartmental water exchange effects. Neuroimage. 2025;314:121258. doi: 10.1016/j.neuroimage.2025.121258 40349743 PMC12270005

[93] Lee H-H, Papaioannou A, Novikov DS, Fieremans E. In vivo observation and biophysical interpretation of time-dependent diffusion in human cortical gray matter. Neuroimage. 2020;222:117054. doi: 10.1016/j.neuroimage.2020.117054 32585341 PMC7736473

[94] Olesen JL, Østergaard L, Shemesh N, Jespersen SN. Diffusion time dependence, power-law scaling, and exchange in gray matter. Neuroimage. 2022;251:118976. doi: 10.1016/j.neuroimage.2022.118976 35168088 PMC8961002

[95] Lasič S, Just N, Nilsson M, Szczepankiewicz F, Budde M, Lundell H. Spectral principal axis system (SPAS) and tuning of tensor-valued encoding for microscopic anisotropy and time-dependent diffusion in the rat brain. Imaging Neurosci (Camb). 2025;3:IMAG.a.35. doi: 10.1162/IMAG.a.35 40800797 PMC12319979

[96] Szczepankiewicz F, Molendowska M, Lasič S, Safi ME, Gottschalk M, Sereti E. Restriction-weighted q-space trajectory imaging (ResQ): toward mapping diffusion time effects with tensor-valued diffusion encoding in human prostate cancer xenografts. bioRxiv. 2025. doi: 10.64898/2025.12.08.692924 PMC13311205 42366513

[97] Fischl B, Dale AM. Measuring the thickness of the human cerebral cortex from magnetic resonance images. Proc Natl Acad Sci U S A. 2000;97(20):11050–5. doi: 10.1073/pnas.200033797 10984517 PMC27146

[98] Vis G, Nilsson M, Westin C-F, Szczepankiewicz F. Accuracy and precision in super-resolution MRI: Enabling spherical tensor diffusion encoding at ultra-high b-values and high resolution. Neuroimage. 2021;245:118673. doi: 10.1016/j.neuroimage.2021.118673 34688898 PMC9272945

