# Supplementary material for: Analysis of cortical dysplasias using b-tensor encoding diffusion MRI in an animal model
Source: PLoS One. 2026 Apr 3;21(4):e0346132. doi: 10.1371/journal.pone.0346132 (PMC13048379; doi:10.1371/journal.pone.0346132)
Supplement: Attachment — Submitted filename: response.pdf [file pone.0346132.s004.pdf]

Response to second round of comments.

Reviewer 2

I am very satisfied with the review and recommend the paper for publication. I have a few comments that the authors can consider, if they wish, without me needing to see the response.

We thank Reviewer 2 for the opportunity to clarify the following two details.

1. Please check that the translation from Ki and Ka to Kbulk and Kshear is correct. For example, Ka is not equal to Kshear so there should be some factor included that may require extra attention in case this was not done. So please check if you actually intended to use Kμ? I have not visited those calculations in some time, so take this with a grain of salt, but from a quick look at the papers and code I thought it worth warning the authors.

The metrics provided by QTI+ are Kshear and Kbulk, which is what we report in this work. In the first version of the manuscript we had changed their names to Ka and Ki to (in our mind) facilitate their interpretation. We now see that this adds to the already complicated collection of names for similar diffusion metrics and have decided to retain Kshear and Kbulk.

In addition, acknowledging that Kμ and Kshear are adjacent but not identical metrics, we checked our data and, as expected, found them to have a very high correlation (r=0.996), making the addition of Kμ redundant.

2. The description of Kshear is somewhat off ("Kshear informs of the variance in microscopic anisotropy"). It suggests that a voxel that is described by many identical domain diffusion tensors (all are equally anisotropic but have different orientation such that the variance in anisotropies is zero) should have a Kshear that is zero. But this is not what Kshear or Kμ measure. Please check/rephrase.

We agree that our previous interpretation lacked the contribution of orientational variance. This is now included, with the phrase reading:

> K_{shear} informs of the variance in microscopic anisotropy **and dispersion of microenvironments** (i.e., tensor shapes **and orientations**), and helps separate tissue components by their geometry (e.g., axonal from non-axonal components).
